# Antiangiogenic agents after first line and sorafenib plus chemoembolization: a systematic review

**DOI:** 10.18632/oncotarget.19449

**Published:** 2017-07-22

**Authors:** Andrea Casadei Gardini, Daniele Santini, Giuseppe Aprile, Nicola Silvestris, Emanuele Felli, Francesco Giuseppe Foschi, Giorgio Ercolani, Giorgia Marisi, Martina Valgiusti, Alessandro Passardi, Marco Puzzoni, Marianna Silletta, Oronzo Brunetti, Giovanni Gerardo Cardellino, Giovanni Luca Frassineti, Mario Scartozzi

**Affiliations:** ^1^ Department of Medical Oncology, Istituto Scientifico Romagnolo per lo Studio e Cura dei Tumori (IRST) IRCCS, Meldola, Italy; ^2^ Medical Oncology Department, University Campus Bio-Medico, Via Álvaro del Portillo, Rome, Italy; ^3^ Department of Medical Oncology, University Hospital, Udine, Italy; ^4^ Medical Oncology Unit, National Cancer Research Centre, Istituto Tumori “Giovanni Paolo II”, Bari, Italy; ^5^ Hôpital Hautepierre Service de Chirurgie Générale, Hépatique, Endocrinienne et Transplantation Université de Strasbourg, Strasbourg, France; ^6^ DPT Internal Medicine, Faenza Hospital, Faenza, AUSL Romagna, Forli, Italy; ^7^ Department of General Surgery, Morgagni-Pierantoni Hospiatal, AUSL Romagna, Forli, Italy; ^8^ Department of Medical and Surgical Sciences, University of Bologna, Bologna, Italy; ^9^ Biosciences Laboratory, IRST IRCCS, Meldola, Italy; ^10^ Department of Medical Oncology, University Hospital Cagliari, Cagliari, Italy

**Keywords:** hepatocellular carcinoma, tace, antiangiogenic, second line, transcatheter arterial chemoembolization

## Abstract

Transarterial chemoembolization (TACE) is the standard treatment for intermediate stage, although the combination of TACE with sorafenib may theoretically benefit HCC patients in intermediate stage. Owing to the significant antiangiogenic effect of sorafenib and the limitation of TACE, it is rational to combine them. Though the strategy of combining TACE and sorafenib has been increasingly used in patients with unresectable HCC but the current evidence is controversial and its clinical role has not been determined yet.

In first-line therapy, patients receiving sorafenib had increased overall survival and progression free survival. Therefore several antiangiogenic agents have entered clinical studies on HCC, many with negative results. This review discusses the current drug development for patients with HCC and role of TACE plus sorafenib.

## INTRODUCTION

Hepatocellular carcinoma (HCC) is the sixth cause of cancer in the world [1].

TACE is the best treatment of intermediate stage in HCC in Europe and the United States [1-2] but in many Asian countries it’s also used in selected cases of advanced disease [3-6].

Despite high local disease control rate and the possibility to improve patients’ survival, TACE is considered a palliative procedure [7, 8].

Hypoxia caused by TACE can leads to the local release of angiogenic growth factors, this release may contribute to tumor recurrence or metastases and poor outcome [9-12].

Sorafenib acts through inhibition of vascular and endothelial growth factor receptor 2 and 3 and platelet-derived growth factor receptor beta leading to an anti-angiogenic effect. Molecular predictors of sorafenib efficacy have not yet been identified [13-17].

Therefore, the combination of TACE with sorafenib may theoretically benefit HCC patients.

Currently, sorafenib has been shown to significantly increase overall survival (OS) and progression free survival (PFS) in patients with BCLC stage C.

Hepatocellular carcinoma are vascular tumours and inhibition of angiogenesis may be represent a potential therapeutic target. For this reason, several antiangiogenic agents have studies on HCC.

This review discusses the current drug development for patients with HCC and the combination of TACE plus sorafenib.

## MATERIALS AND METHODS

### Search strategy and selection criteria

**Figure 1:**
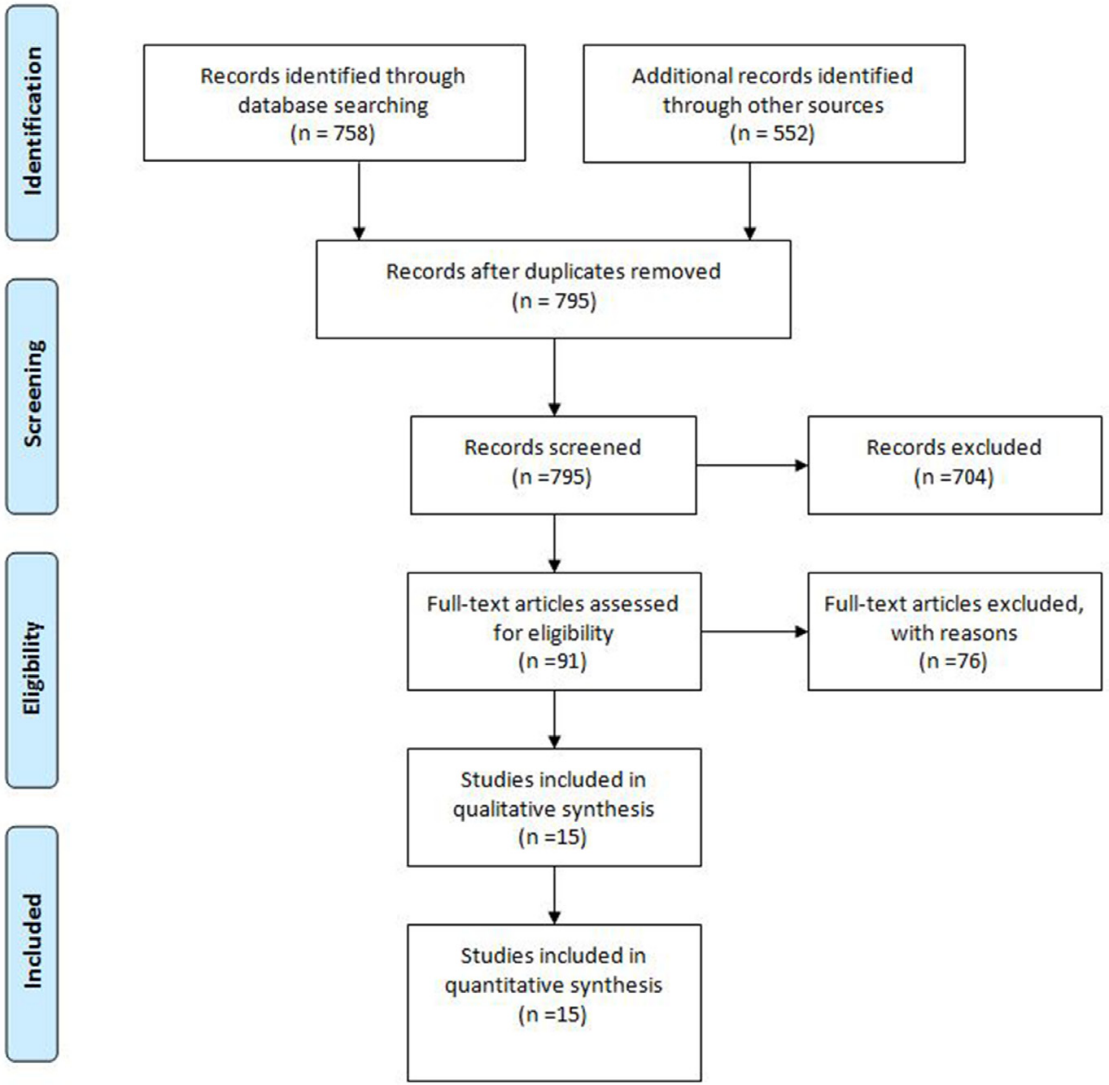
PRISMA guidelines of TACE plus Sorafenib

**Figure 2:**
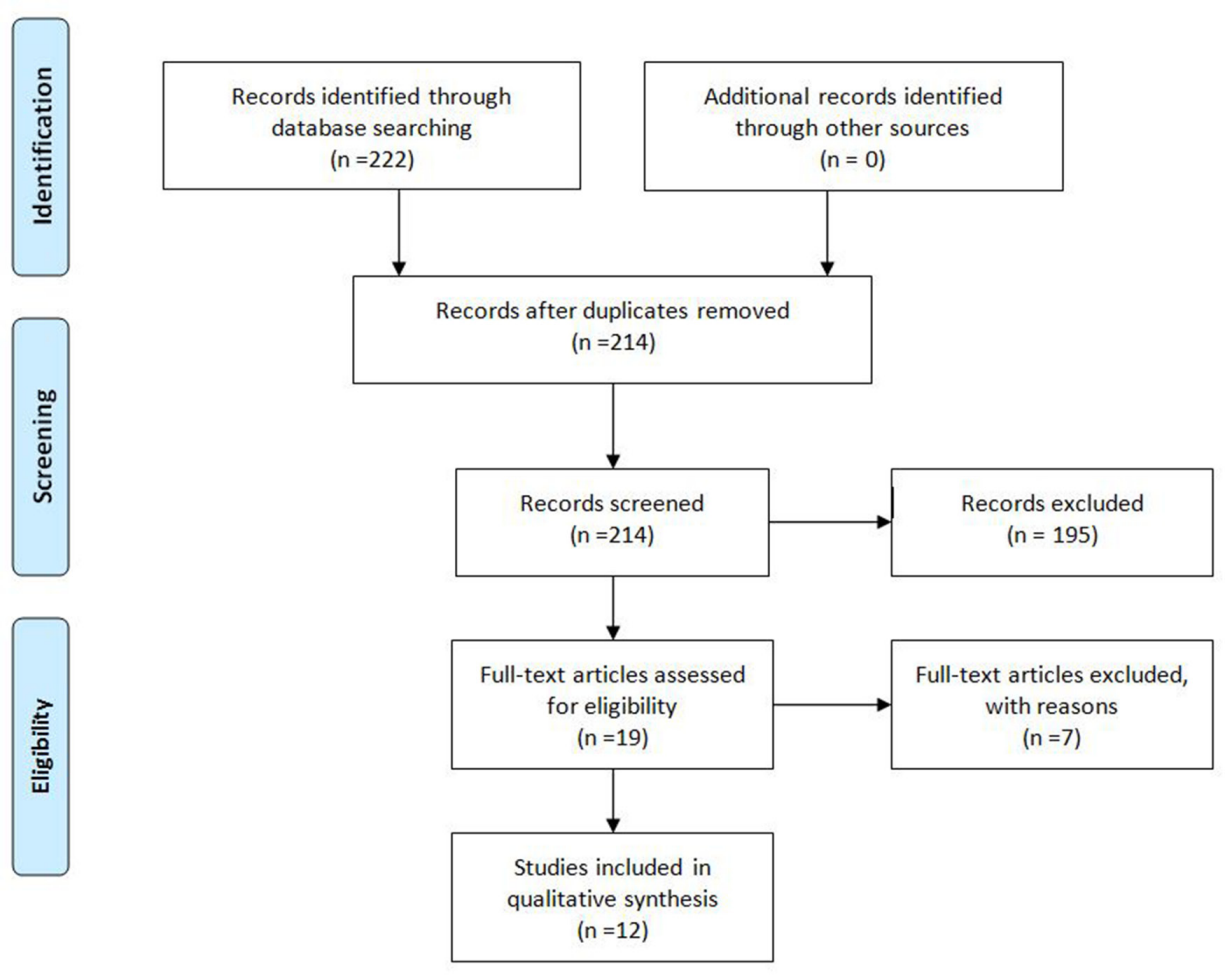
PRISMA guidelines of antiangiogenic agents after Sorafenib

We searched on PubMed/MEDLINE and Clinicaltrials.gov using the following keywords: ‘‘second-line AND hepatocellular carcinoma’’ for antiangiogenic study in second line and “TACE plus Sorafenib’’ or “TACE and Sorafenib” for sorafenib plus TACE. We use PRISMA guidelines (Figures 1 and 2) to identify all study.

We reviewed the references of all included articles to identify additional sources of data, missing articles or meeting abstracts. When multiple sources of data from the same study population were found, we relied on the data from the most complete peer-reviewed publication.

### Sorafenib plus tace

Several studies have explored this combination therapy, from either retrospective series or small early-phase studies. Moreover there is a wide heterogeneity in study design especially in underlying liver disease (HCV vs HBV), overall hepatic function (Child A vs Child B), BCLC stage (A vs B vs C), modality of sorafenib treatment (sequential vs. continuous vs. interrupted), and variables linked to TACE treatment.

Table 1 summarizes the main studies of sorafenib plus TACE.

**Table 1:** summarizes the main studies of sorafenib plus TACE

	Trial name/PI	Results
PHASE III	SPACE Trial	mTTP: (S) 169 d vs. (P) 166 d p<0.072
	Kudo	mTTP: (S) 5.4 m vs. (P) 3.7 m p<0.252
	Sansonno	mTTP: (S) 9.2 m vs. (P) 4.9 m p<0.001
	Hofmann	mTTP: (S) 125 day vs. (P) 171 day m p=0.005
PHASE II	START Trial	mTTP: 9 m ORR: 53.8%
	SOCRATES Trial	mTTP: 16.4 m
	COTSUN Trial	mTTP: 7.1 m

mTTP: median time to progression; d: days; m: months; (S): sorafenib; (P): placebo; ORR: overall response rate.

According to the classification by Strebel et al [18], trials that encourage combination of TACE with sorafenib are divided into three main categories:

1. continuous schedule: patients are treated all the time with sorafenib.
2. Interrupted schedule: patients are treated with sorafenib only between TACE sessions.
3. Sequential schedule: the patients was treated with TACE followed by sorafenib.

### Randomized trial

In literature there are four randomized trial.

The SPACE trial, is a phase II randomized trial that enrolled 307 patients [19]. Even if the primary endpoint was no achieved (p= 0.072), in fact the time to progression was similar between two groups (169 days for sorafenib group respect166 days for placebo group). The HR for overall survival was 0.898.

Kudo et al presented a double blind placebo controlled phase III trial. The trial was designed before the indication of sorafenib in first line of advanced HCC [20]. The study enrolled 458 patients. The study failed the primary endpoint, the median time to progression was of 5.4 in the sorafenib group and 3.7 months in placebo group (p = 0.252). Probably, these results may be due to the fact that 91% had dose interruptions and 73% of patients had sorafenib dose reductions.

Sansonno et al, in a Italian single-center randomized study enrolled 62 patients [21]. Patients received sorafenib or placebo after conventional TACE. The trial was positive with a median time to progression of 9.2 months in the sorafenib group vs 4.9 months in the placebo group (p < 0.001).

In the study of Hofmann et al [22], fifty patients were randomly in double-blinded and treated with TACE plus Sorafenib or placebo. The trial was negative, the HR of time to progression was 1.106 (95% CI: 0.387, 3.162). The results was negative also for objective response rate, disease control rate, progression free survival and time to liver-transplant.

Recently, a meta-analysis that included all four studies show that TACE plus sorafenib may have superiority of TACE only in terms of time to progression (HR=0.77, P=0.005) but not for OS (HR=0.97, P=0.828), ORR (RR=1.20, P=0.257) and DCR (RR=1.04, P=0.568) [23].

### Phase II trial

START trial [24] enrolled 192 Asian patients. 81.2% of patients were with HBV. Patients received sorafenib with interrupted schedule (3 days before and after conventional TACE). 52 patients had grade 3/4 adverse events, only 8.1% discontinued sorafenib for toxicities with no treatment-related deaths. Median time to progression was 9 months with a 53.8% of response rate.

SOCRATES trial [25], a multicenter single-arm trial enrolled 43 patients. Median overall survival was 20.1 months with time to progression was 16.4 months. The authors value with EASL criteria the response and showed 74,4% of disease control rate.

COTSUN trial [26], a single-arm enrolled 50 patients with interrupted schedule with median time to progression was 7.1 months, many patients have discontinued sorafenib for thrombocytopenia and skin toxicity.

Another phase II study enrolled 35 patients [27]. The study reported disease control rate of 95% according to RECIST criteria with 40 dose interruptions and 25 dose reductions for sorafenib. In the paper the authors did not report the data of time to progression, progression free survival or overall survival.

### Ongoing study

The SELECT trial [28] is an ongoing prospective multicenter randomized study. Aims of the study was to evaluate efficacy and safety of continued schedule of sorafenib combined with conventional TACE.

The second trial is a phase III randomized trial [29, 30] with intermediate HCC and Child-Pugh score A-B7 with continued schedule. The primary endpoint is progression free survival, secondary endpoints are overall survival and pattern of treatment failure (extrahepatic vs intrahepatic).

The last is an ongoing phase IV non-randomized study [31] that evaluating the benefits of traditional TACE plus sequential sorafenib with sequential schedule (sorafenib started 2-4 weeks after) versus TACE alone in this setting of patients with child-Pugh score A. The primary endpoint is overall survival and secondary endpoint is time to progression.

### Antiangiogenic agents after sorafenib

**Figure 3:**
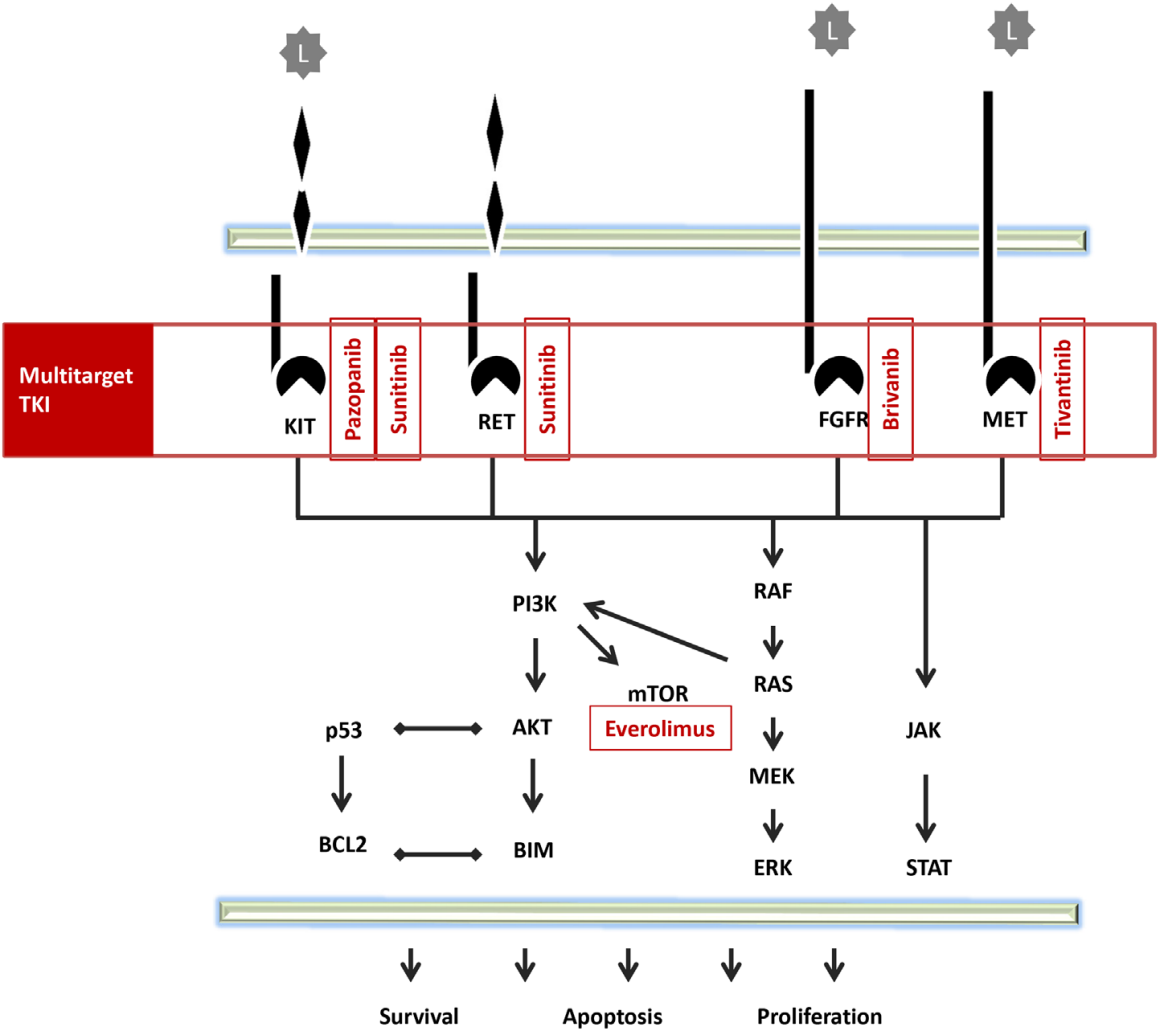
Pathway of multitarget TKI

**Figure 4:**
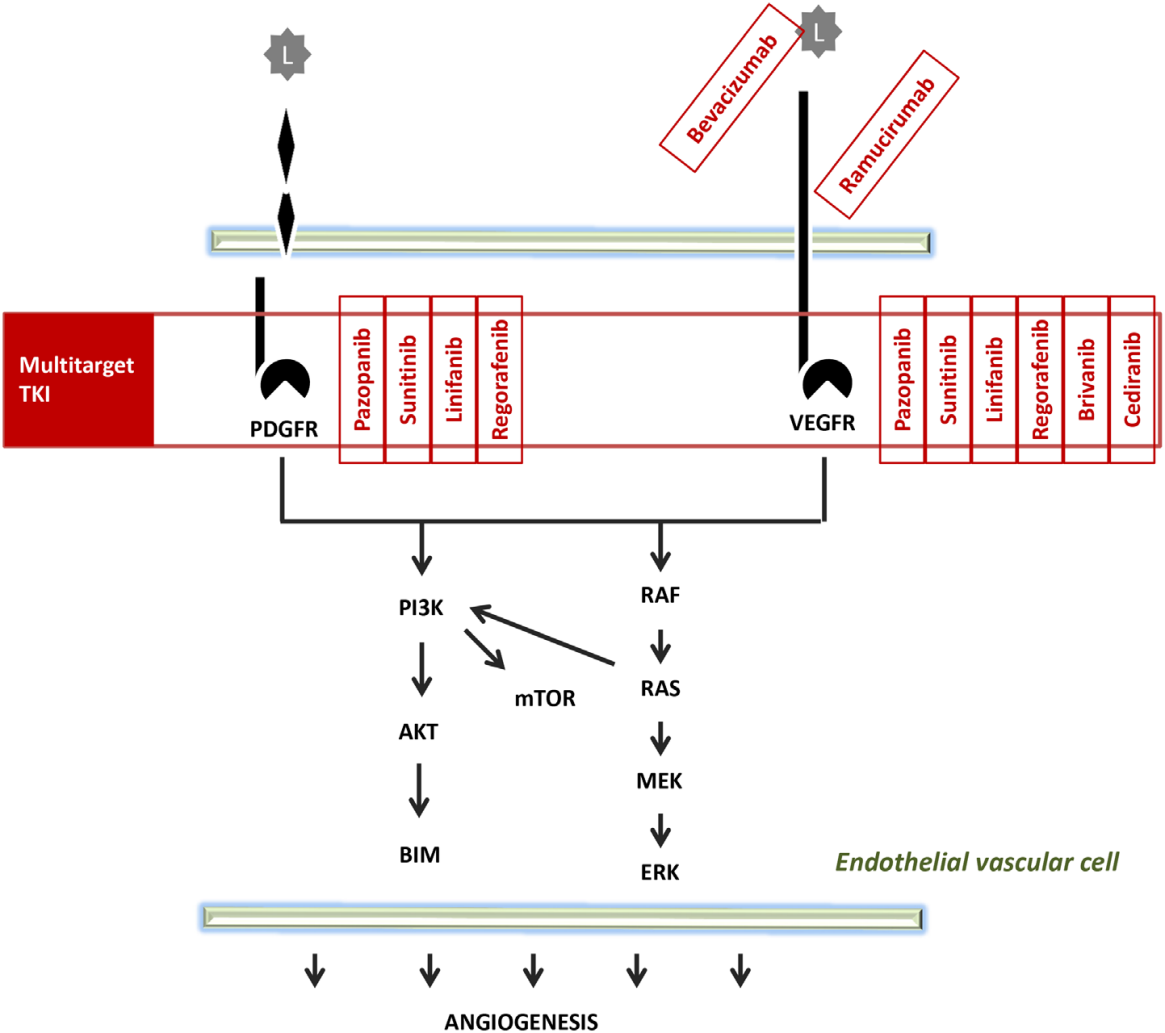
Pathway of multitarget TKI

Several antiangiogenic agents have entered clinical studies in HCC as summarized in Table 2, in Figures 3 and 4 show the pattern of new drug.

**Table 2:** Table 2 drugs on trial and results

Drug	Phase Study	Results
Regorafenib	III	mPFS: 3,1 months mOS: 10,6 months
Bevacizumab	II	mPFS: 6,9 months mOS: 12.4 months
Beva + gemox	II	mOS: 15.0 months
Sunitinib	III	mOS:9,8 months
Sunitinib vs sorafenib	III	mOS: 7,.9 months vs. 10.2 months
Brivanib	II	mOS: 9.7 months
BRISK-PS (Brivanib vs. placebo)	III	mOS: 9.4 months vs. 8.3 months
BRISK-FL (brivanib vs placebo)	III	mOS: 9.4 months vs. 8.3 months
Pazopanib	I	mTTP: 4.5 months
Tivantinib	II	mOS: 7.2 months vs. 3.8 months in patients with high c-met
Dovitinib (dovitinib vs sorafenib)	II	mOS: 8.0 months vs 8.4 months mTTO: 4.1 months vs 4.1months

### Regorafenib

Regorafenib show that can be improved overall survival in HCC patients in the RESORCE trial [32].

In the RESORCE trial a total of 573 patients were randomized (patients were randomized 2:1 to receive regorafenib or placebo) after failure of first line with sorafenib. Regorafenib reduce 38% in the risk of death (p <0.001) and median overall survival was 10.6 months in patients treated with regorafenib versus 7.8 months in patients treated with placebo. Regorafenib reduced by 54% the risk of progression or death with median progression free survival of the patients treated with regorafenib was 3.1 vs 1.5 months for patients treatment with placebo (time to progression was 3.2 vs 1.5 respectivily). Disease controll rate was 65.2% for regorafenib vs 36.1% for placebo (p<0.001).

Actualy regorafenib in second line after progression of Sorafenib is the standard of care.

### Bevacizumab

Several studies have explored the use of bevacizumab as a single agent or in combination with other agents.

Bevacizumab was tested in two study as a single agent. In the first study published by Siegel et al [33], the median progression free survival was 6.9 months and median overall survival was 12.4 months. In the second study [34] 13.9% patients on 43 had partial response and 42% had disease control rate at 16 weeks.

Bevacizumab was tested in combination with erlotinib in two phase II studies [35, 36].

Kaseb et al. [35] enrolled 59 patients. 24% achieved partial response, 56% had stable disease, 10% progressed (overall survival was 13.7 months and PFS was 7.2 months). In the second study Yau et al [36] enrolled 10 patients with median time to progression of 1.81 months and overall survival was 4.37 months.

In conclusion, these studies demonstrated evidence of antitumor activity of Bevacizumab in HCC. Future prospective studies will tell us the real effectiveness of this drug.

### Sunitinib

Cheng et al. [37] completed a randomized phase III trial comparing sunitinib with sorafenib.

1,074 patients were randomized with median overall survival of 7.9 in the sunitinib arm vs. 10.2 months in the sorafenib arm (P=0.0014).

For these reasons the authors concluded that overall survival with sorafenib was significantly higher than with sunitinib.

### Brivanib

Brivanib is a tyrosine kinase inhibitor that shows selective inhibition of VEGFR and fibroblast growth factor receptor.

The antitumor activity of brivanib in patients with HCC was tested in three phase III studies.

In the BRISK-FL study [38] was tested in first-line. Median overall survival, time to progression and disease control rate were similar between sorafenib and brivanib.

In the BRISK-PS study [39] brivanib was tested in 395 patients with advanced HCC who progressed or were intolerant to sorafenib. Median overall survival was similar between brivanib and placebo arms (9.4 months for brivanib and 8.2 months for placebo, HR 0.89; P = .3307), but brivanib was associated with a longer median time to progression than sorafenib (4.2 months for brivanib and 2.7 months for placebo HR: 0.56 P < .001).

The last (BRISK-APS) is an ongoing study [40] enrolling second-line patients from the Asian-Pacific region.

### Linifanib

Linifanib is an orally active, potent and selective inhibitor of VEGFR and PDGFR.

A phase III trial [41] randomized 1,035 patients with median overall survival of 9.1 months in the linifanib arm and 9.8 months in the sorafenib arm. Linifanib and sorafenib resulted in similar overall surival associated with more toxicity for linifanib arms.

### Pazopanib

Pazopanib is an orally inhibitor of VEGFR, PDGFR and c-Hit.

A phase I dose-escalating study of pazopanib [42] was conducted to determine the maximum tolerated dose, it was defined at 600 mg once daily. No phase II trial has been programmed.

### Tivantinib

Tivantinib is a selective inhibitor of MET.

In a phase II trial [43] 107 patients with unresectable HCC progressed after first-line treatment or were intolerant. Patients were randomized to receive either 360 mg or 240 mg of tivantinib twice daily or placebo.

Median OS was 7.2 months for patients treated with tivantinib compared with 3.8 months for patients treated with placebo (HR: 0.38, p = 0.01). Median time to progression was 2.9 months for patients treated with tivantinib compared 1.5 months for patients treated with placebo (HR 0:43, p = 0.03). In this study MET level was predictive of time to progression and overall survival.

A global randomized phase III study comparing the clinical activity of tivantinib versus best supportive care in patients with hepatocellular carcinoma in second-line therapy will be presented at ASCO 2017 but a press release announced that the study did not meet the primary endpoint.

### Cabozantinib

Cabozantinib is a dual MET/VEGFR-2 inhibitor. It was tested in second-line therapy for hepatocellular carcinoma in a phase II randomized study [44], with 41 patients enrolled, 4.4 months median PFS and 15.1 months median overall survival. A global randomized phase III study is ongoing.

### Ramucirumab

A phase II study [45] of 42 patients with advanced hepatocellular carcinoma showed that ramucirumab in first-line produced a 50% DCR and a median PFS of 4.3 months.

In the REACH study thee results show that the HR for overall survival was 0.866 (p = 0.1391); overall survival was 9.2 months and progression free survival was 2.8 months for the ramucirumab arm versus 7.6 months for overall surivival and 2.1 months for progression free survival in the placebo arm.

A subgroup analysis show that patients with high baseline alpha-fetoprotein (≥400 ng/mL) have a median OS of 7.8 months for ramucirumab versus 4.2 months for placebo, HR was 0.67 (p = 0.0059) with [46].

This suggests that elevated baseline AFP may be a predictive marker for survival benefit to ramucirumab, and it is now the basis for a biomarker selected Phase III study (REACH-2) in the same setting.

### Cediranib

Cediranib and vatalanib are oral inhibitors of VEGF receptor tyrosine kinase. A phase II study was published [47] with cediranib, but with no responses to the drug. Median OS was 5.8 months. No phase III trial has been programmed.

### Dovitinib

Dovinitib is a potent tyrosine kinase inhibitor targeting at FGFR, VEGFR, PDGFR and c-kit.

In preclinical study dovinitib showed an activity in HCC [48].

A randomized phase II study [49] comparing the clinical activity of dovitinib versus sorafenib. In this trial dovitinib and sorafenib resulted in similar OS and TTP. No phase 3 trial has been planned.

## CONCLUSIONS

Over the last decade, the progress in managing advanced liver cancers has been slow for three many reasons. First of all, we have probably failed in the planning phase of clinical trials. Published data show a wide heterogeneity in the study design, ethnicity of patients enrolled, underlying liver disease (HCV vs. HBV), grade of liver decompensation (Child A vs. Child B), BCLC stage (A vs. B vs. C often mixed in variable proportions), presence of patients with vascular invasion or metastatic disease, modality of sorafenib treatment (sequential vs. continuous vs. interrupted), type of TACE (conventional vs DEB-TACE), type of chemotherapy, number of drugs delivered (single agent vs. combination), dose of chemotherapy, use of embolizing materials, number of procedures administered, and schedule (on-demand vs. pre-planned). Consequently, the results of the studies are contrasting. Secondly, we lack effective drug. Thirdly, we did not properly plan effective strategies with treatment combinations or sequences. Combination therapy may provide clinical benefit for patients with HCC but subjects selection is imperative to avoid unacceptable toxicities or even harm (for example liver decompensation).

A better understanding of the biology of HCC, the variables that could predict response to sorafenib, and the mechanisms by which anti-angiogenic agents work in concert with conventional chemotherapeutic agents used in TACE, will be critical to design the definitive trial on the combination treatment. Post hoc analysis of the SHARP trial show that low baseline concentrations of angiopoietin-2 and VEGF-A were associated with better OS [50, 51]. Casadei Gardini et al and Scartozzi et al have studied the role of polymorphisms and response to sorafenib. This two study showed that *VEGF-A, VEGF-C and eNOS* polymorphisms were independent factors of PFS and OS [52, 53].

We probably would have different therapeutic options in the first and second line of HCC treatment in the next five years, changing the scenario for this disease. As previously reported in the latest years for the kidney tumor we could have instead of few therapeutic options several active compounds.

Many promising drugs in phase II studies failed in phase III trials. There are multiple reasons for these failures. The most important is the faulty study designs, in particular stratification at the time of randomization.

In the future we have to identify new meccanism of hepatocarcinogenesis to study new treatment approach.
